# Supplementary material for: Exploring the interior of 3D endoluminal lesions in the air spaces by a novel electronic biopsy technique: A preliminary study of endoluminal colon lesions
Source: PLoS One. 2023 May 11;18(5):e0284142. doi: 10.1371/journal.pone.0284142 (PMC10174533; doi:10.1371/journal.pone.0284142)
Supplement: Attachment — Submitted filename: rebuttal_PlOS.docx [file pone.0284142.s001.docx]

PONE-D-21-25670

Exploring the interior of 3D endoluminal lesions in the air spaces by a novel electronic biopsy technique: a preliminary study of endoluminal colon lesions and comparison with traditional surface rendering method

PLOS ONE

Dear Dr. Chen,

Thank you for submitting your manuscript to PLOS ONE. After careful consideration, we have decided that your manuscript does not meet our criteria for publication and must therefore be rejected.

Specifically:

I am sorry that we cannot be more positive on this occasion, but hope that you appreciate the reasons for this decision.

Yours sincerely,

Amita Nandal

Academic Editor

PLOS ONE

Additional Editor Comments:

First of all, we would like to thank the anonymous reviewer for giving the constructive suggestions to improve this manuscript. The responses for the comments are summarized in the following.

The paper is yet not ready for publishing in any Journal. It is in raw form.

Results are weak without any comparison with state of art.

Response:

**Clinical evaluation**

Page 13, the last aragraph: EB preserved the 3D spatial shape, gray-level pattern and images correlation of the endoluminal lesions during the biopsy procedure (Table 1) as previous described GM or TGM methods [5, 8]. EB scored significantly higher than did SR in the criteria evaluated by the two radiologists, regardless of the malignant or benign status as determined pathologically (one-tailed Wilcoxon signed ranks test, P < 0.01). According to the localization and gray level correspondence, EB images provided a significantly higher correlation of 3D to 2D images than did SR images (one-tailed Wilcoxon signed ranks test, P < 0.01) (Table 1). In the evaluation of the rendering effect of the new surface after each biopsy, EB images reveal increased gray level pattern (texture) changes with statistically difference compared with GM or TGM methods (P<0.01). No statistical difference of the spatial and correlation effect between the EB and GM/ TGM.

**Table 1. Image features and classification scores of endoluminal lesions on CTC.**

| **Lesion Class** | **Case number** | **Rated feature** | **SR** | **GM** | **TGM** | **EB** | **P ***  **vs. SR** |
| --- | --- | --- | --- | --- | --- | --- | --- |
| **Potential malignancy** | 34 | Spatial shape | 2 | 2.44 | 3.00 | 3 | <0.01 |
|  |  |  | 2 | 2.97 | 2.97 | 2.94 |  |
|  |  | texture | 1 | 2.03 | 2.44 | 2.98 | <0.01 |
|  |  |  | 1 | 2.29 | 2.32 | 2.92 |  |
|  |  | Correlation | 1.9 | 2.47 | 2.94 | 3 | <0.01 |
|  |  |  | 1.92 | 2.94 | 2.94 | 2.94 |  |
| **Benign** | 28 | Spatial shape | 2 | 2.46 | 2.96 | 3 | <0.01 |
|  |  |  | 2 | 2.93 | 2.93 | 2.88 |  |
|  |  | texture | 1 | 2.07 | 2.5 | 2.99 | <0.01 |
|  |  |  | 1 | 2.32 | 2.43 | 2.73 |  |
|  |  | Correlation | 1.31 | 2.00 | 2.67 | 2.41 | <0.01 |
|  |  |  | 1.88 | 2.54 | 2.64 | 2.76 |  |

Potential malignancy: polyps or tumors

Benign lesions: stool, fluid feces, ileocecal valves, intraluminal calcification

* Wilcoxon signed ranks test (one-tailed)

Upper and lower sub-rows of every rated parameter indicate the average score of two different radiologists.

**Diagnostic Performance**

Page 15, the first paragraph: Performance studies showed EB retains high sensitivity to predict potential malignancies as GM or TGM methods (Table 2) [5, 8]. Using TGM or EB to investigate deeper layers of a lesion increased the sensitivity to 100%. The major disadvantages of SR are its low specificity of benign lesions (14.3–17.9%) and low diagnostic accuracy, leading to no significant improvement in diagnostic performance (P = 0.082). GM or TGM has known to significantly increase the specificity and diagnostic accuracy, yielding significantly improved diagnostic performance in comparison with SR [5, 8]. The results are further improved if deeper layers are imaged by EB (specificity, from 75.0-85.7% to 89.3-92.9%; diagnostic accuracy, from 88.7-93.5% to 95.2-96.8%) (Table 2).

**Table 2. Diagnostic performance of SR and EB**

| **Reviewer/ display method** | **Sensitivity**  **(95% CI)** | **Specificity**  **(95% CI)** | **Diagnostic**  **accuracy** | **P *** |
| --- | --- | --- | --- | --- |
| **Reader 1** |  |  |  |  |
| **SR** | 97.1%  (0.851; 0.995) | 17.9%  (0.079; 0.356) | 61.3% | 0.082 |
| **GM** | 100%  (0.899,1) | 64.3%  (0.458,0.793) | 83.9% | <0.001 |
| **TGM** | 100%  (0.899,1) | 85.7%  (0.685,0.943) | 93.5% | <0.001 |
| **EB** | 100%  (0.899; 1) | 92.9%  (0.774; 0.980) | 96.8% | <0.001 |
| **Reader 2** |  |  |  |  |
| **SR** | 88.2%  (0.734; 0.953) | 14.3%  (0.057; 0.315) | 54.8% | 1.000 |
| **GM** | 97.1%  (0.851,0.995) | 53.6%  (0.358,0.705) | 77.4% | <0.001 |
| **TGM** | 100%  (0.899,1) | 75.0%  (0.566,0.873) | 88.7% | <0.001 |
| **EB** | 100%  (0.899; 1) | 89.3%  (0.728; 0.963) | 95.2% | <0.001 |

CI, confidence interval

*Values represent comparison between final pathology and diagnostic performance of SR and EB

There is no literature reviewed .

Response:

Page 4, the second paragraph :

Despite the usefulness of CTE, the traditional surface rendering (SR) technique used in this technology has two drawbacks. First, SR converts the data into a simplified, binary form for surface area estimation [4]. Since no gray-level information for the endoluminal lesion surface is visible, the specificity and diagnostic accuracy for imaging intra- and endoluminal lesions using only SR is poor [5]. Second, SR only evaluates the surface information of 3D endoluminal lesions. To facilitate the discrimination and management of such lesions, the exploration of gray-level changes below the surface of endoluminal lesions warrants further study.

Volume rendering (VR) is also widely used in CTE to distinguish between true and false polyps. On a typical monochromatic volume-rendered 3D endoluminal view, a polyp cannot be distinguished from polyp-like tagged stool without reference to translucency maps [6]. Translucency mapping requires the assignment of blue, green, red, and white color channels to areas of increasing attenuation [7], a process that is subjective and influenced by user settings.

Chen et al. report a GM technique that modifies the traditional surface rendering images by applying gray level to the surface points in CTC [5]. The technique is reported to be more accurate than the traditional surface rending method in correlating CTC and 2D views, thereby improving the identification and differentiation of endoluminal lesions. In contrast to the abstract color transfer function used in VR, the gray-level range and character mapped in Chen’s technique are the same as those in the original CT images, making them easier for radiologists and physicians to interpret.

To speed up the navigation process and highlight gray-level patterns in endoluminal lesions, Chen et al. also developed a target gray-level mapping (TGM) technique [8]. Users can select any region of interest for further 3D analysis by this method. However, both the GM and TGM techniques only survey gray-level information from the surface points of a 3D object. Radiologists are still unable to visualize tissues below the surface in the endoluminal view to investigate a suspicious lesion.

To help users evaluate gray-level information in 3D endoluminal lesions below the surface, this study investigates an electronic biopsy method that would allow for further exploration and differentiation of such lesions. Similar to the endoscopic biopsy, the purpose of the study is the internal exploration of 3D endoluminal lesions using an EB method. CTC cases were used as an example to demonstrate the 3D shape, texture effect, image correlation of the EB method and its impact on clinical diagnosis. This study may be extended to the endoluminal lesions in other air filled spaces, such as airway and upper gastrointestinal systems.

References:

1.	Siegel RL, Miller KD, Fedewa SA, Ahnen DJ, Meester RGS, Barzi A, et al.

Colorectalcancer statistics, 2017. CA Cancer Journal for Clinicians. 2017;67:177–193.

2.	Silvis SE, Nebel O, Rogers G, Sugawa C, Mandelstam P. Endoscopic complications. Results of the 1974 American Society for Gastrointestinal Endoscopy Survey. JAMA. 1976;235(9):928-930

3.	Dominitz, J. A., Eisen, G. M., Baron, T. H., Goldstein, J. L., Hirota, W. K., Jacobson, B. C., et al. Complications of colonoscopy. Gastrointestinal endoscopy. 2003;57(4):441-445

4.	Heath DG, Soyer PA, Kuszyk BS et al. Heath, D. G., Soyer, P. A., Kuszyk, B. S., Bliss, D. F., Calhoun, P. S., Bluemke, D. A., et al. Three-dimensional spiral CT during arterial portography: comparison of three rendering techniques. Radiographics. 1995;15(4):1001-1011

5.	Chen, L. S., Hsu, T. W., Chen, S. J., Chang, S. H., Lin, C. W., Chen, Y. R., et al. Application of gray level mapping in computed tomographic colonography: a pilot study to compare with traditional surface rendering method for identification and differentiation of endoluminal lesions. Br J Radiol. 2017; 90:20160733

6.	Park, S. H., Lee, S. S., Kim, J. K., Kim, M. J., Kim, H. J., Kim, S. Y., et al. Volume rendering with color coding of tagged stool during endoluminal fly-through CT colonography: effect on reading efficiency. 2008;Radiology 248:1018-1027

7.	Pickhardt PJ. Translucency rendering in 3D endoluminal CT colonography: a useful tool for increasing polyp specificity and decreasing interpretation time. AJR Am J Roentgenol. 2004;183:429-436

8.	Chen, L. S., Hsu, T. W., Chen, S. J., Chang, S. H., Lin, C. W., Chen, Y. R., et al. Improving Image Correlation and Differentiation of 3D Endoluminal Lesions in the Air Spaces Using a Novel Target Gray Level Mapping Technique: A Preliminary Study of Its Application to Computed Tomographic Colonography and Comparison with Traditional Surface Rendering Method. Journal of medical and biological engineering. 2020;40(6):868-879.

9.	Liedenbaum, M. H., De Vries, A. H., Gouw, C. I. B. F., Van Rijn, A. F., Bipat, S., Dekker, E., et al. CT colonography with minimal bowel preparation: evaluation of tagging quality, patient acceptance and diagnostic accuracy in two iodine-based preparation schemes. European radiology. 2010;20(2): 367-376.

10.	Valev V, Wang G, Vannier MW. Techniques of CT colonography (virtual colonoscopy). Crit Rev Biomed Eng. 1999;27:1-25

11.	Lorensen WE, Cline HE. Marching cubes: A high resolution 3D surface construction algorithm. ACM Siggraph Computer Graphics. 1987;21:163–169

12.	Preim B, Botha CP. Visual computing for medicine: theory, algorithms, and applications. Newnes; 2013.

13.	Cotton, P. B., Durkalski, V. L., Pineau, B. C., Palesch, Y. Y., Mauldin, P. D., Hoffman, B., et al. Computed tomographic colonography (virtual colonoscopy): a multicenter comparison with standard colonoscopy for detection of colorectal neoplasia. JAMA. 2004;291:1713-1719

14.	Rockey, D. C., Paulson, E., Niedzwiecki, D. E., Davis, W., Bosworth, H. B., Sanders, L., et al. Analysis of air contrast barium enema, computed tomographic colonography, and colonoscopy: prospective comparison. Lancet. 2005;365:305-311

15.	Pickhardt, P. J. Screening CT colonography: how I do it. American Journal of Roentgenology. 2007;189(2):290-298.

16.	Dachman AH, Laghi A. Atlas of virtual colonoscopy. Springer Science & Business Media, New York; 2011.

17.	Guerrisi, A., Marin, D., Laghi, A., Di Martino, M., Iafrate, F., Iannaccone, R., et al. Diagnostic accuracy of translucency rendering to differentiate polyps from pseudopolyps at 3D endoluminal CT colonography: a feasibility study. La radiologia medica. 2010;115(5), 758-770.

18.	Porter, T., & Duff, T. Compositing digital images. In Proceedings of the 11th annual conference on Computer graphics and interactive techniques. 1984; January, 253-259.
