# Supplementary material for: Exploring the interior of 3D endoluminal lesions in the air spaces by a novel electronic biopsy technique: A preliminary study of endoluminal colon lesions
Source: PLoS One. 2023 May 11;18(5):e0284142. doi: 10.1371/journal.pone.0284142 (PMC10174533; doi:10.1371/journal.pone.0284142)
Supplement: Attachment — Submitted filename: Response to Reviewers.docx [file pone.0284142.s002.docx]

First of all, we would like to thank the anonymous reviewer for giving the constructive suggestions to improve this manuscript. The responses for the comments are summarized in the following.

Reviewer #1: Review Report

• In this paper, the authors aimed to investigate the application of an ‘electronic biopsy’ (EB) technique to computed tomographic colonography (CTC).

• Based on a retrospective analysis of 30 patients with 62 endoluminal lesions of various types, the authors conclude

• The paper is interesting, well structured, and correctly organized. The authors have clearly worked hard to detail their study, but I have some comments:

POINTS OF WEAKNESS

1. Retrospective design.

Response:

Page 24, the second paragraph: This study has limitations. A retrospective study of a small number of pre-selected images in this preliminary study. However, the very small p (<0.01) and high power (>0.8) values warrant the adequate sample size to demonstrate the advantage of EB technique.

2. Small sample size.

Response:

Page 24, the second paragraph: This study has limitations. A retrospective study of a small number of pre-selected images in this preliminary study. However, the very small p (<0.01) and high power (>0.8) values warrant the adequate sample size to demonstrate the advantage of EB technique.

SPECIFIC COMMENTS

1. The type of study and the number of included patients should be mentioned in the abstract.

Response:

Abstract, line 4: A retrospective study of sixty-two various endoluminal lesions from thirty patients (13 males, 17 females; age range, 31 to 90 years) was approved by our institutional review board and evaluated.

2. A flow chart of the study is needed.

Response:

Page 7, the first paragraph: The flow chart methodology of this research is shown in the figure 1.

Data acquisition

Segmentation

Finding the object surface points and

surface rendering

Clinical evaluation & diagnostic performance

Gray level mapping of ROI

Choosing the ROI

Electronic biopsy: ROI erosion and gray-level mapping

Fig 1. Flow chart of methodology.

3. What was the power of sample size calculation?

Response:

Page 15, the second paragraph: EB scored significantly higher than did SR in the criteria evaluated by the two radiologists, regardless of the malignant or benign status as determined pathologically (one-tailed Wilcoxon signed ranks test, P < 0.01). The power values of the paired tests are also high (> 0.8). According to the localization and gray level correspondence, EB images provided a significantly higher correlation of 3D to 2D images than did SR images (one-tailed Wilcoxon signed ranks test, P < 0.01) with high power above 0.8 (Table 1).

**Table 1. Image features and classification scores of endoluminal lesions on CTC.**

| **Lesion Class** | **Case number** | **Rated feature** | **SR** | **GM** | **TGM** | **EB** | **P ***  **vs. SR** | **Agreement**  **EB vs. SR** |
| --- | --- | --- | --- | --- | --- | --- | --- | --- |
| **Potential malignancy** | 34 | Spatial shape | 2 | 2.44 | 3.00 | 3 | <0.01 | 33(97%) |
|  |  |  | 2 | 2.97 | 2.97 | 2.94 |  |  |
|  |  | texture | 1 | 2.03 | 2.44 | 2.98 | <0.01 | 34(100%) |
|  |  |  | 1 | 2.29 | 2.32 | 2.92 |  |  |
|  |  | Correlation | 1.9 | 2.47 | 2.94 | 3 | <0.01 | 33(97%) |
|  |  |  | 1.92 | 2.94 | 2.94 | 2.94 |  |  |
| **Benign** | 28 | Spatial shape | 2 | 2.46 | 2.96 | 3 | <0.01 | 26(93%) |
|  |  |  | 2 | 2.93 | 2.93 | 2.88 |  |  |
|  |  | texture | 1 | 2.07 | 2.5 | 2.99 | <0.01 | 28(100%) |
|  |  |  | 1 | 2.32 | 2.43 | 2.73 |  |  |
|  |  | Correlation | 1.31 | 2.00 | 2.67 | 2.41 | <0.01 | 27(96%) |
|  |  |  | 1.88 | 2.54 | 2.64 | 2.76 |  |  |

Potential malignancy: polyps or tumors

Benign lesions: stool, fluid feces, ileocecal valves, intraluminal calcification

* Wilcoxon signed ranks test (one-tailed)

Upper and lower sub-rows of every rated parameter indicate the average score of two different radiologists.

The values of power of all paired tests (EB vs. SR) are above 0.8.

4. More details about CT parameters are needed

Response:

Page 7, the second paragraph: Helical CT scans of the prone and supine positions were performed 30 seconds after intravenous contrast administration with the following parameters: collimation 64×0.6 mm; gantry rotation time 0.8 s; X-ray Tube Current: 140 mA; Exposure Time: 800 ms; kVp 120. Scanning was performed craniocaudally during a single breath-hold. With the advance of multiple-slice CT, the slice thickness of volume data can be reconstructed to 1.25 mm. All scans were performed using a 64-slice GE Lightspeed VCT scanner (GE HealthCare, Milwaukee, Wisconsin, USA).

5. Why did the authors not perform interviewer agreement to strengthen their results?

Response:

Page 15, the second paragraph: The classifications performed by both observers were in very good agreement—that is, better EB experience than SR (Table 1).

--------------------------------------------------------------------------------

6. PLOS authors have the option to publish the peer review history of their article (what does this mean?). If published, this will include your full peer review and any attached files.

If you choose “no”, your identity will remain anonymous but your review may still be made public.

Do you want your identity to be public for this peer review? For information about this choice, including consent withdrawal, please see our Privacy Policy.

Reviewer #1: Yes: Mohammad Abd Alkhalik Basha

--------------------------------------------------------------------------------

[NOTE: If reviewer comments were submitted as an attachment file, they will be attached to this email and accessible via the submission site. Please log into your account, locate the manuscript record, and check for the action link "View Attachments". If this link does not appear, there are no attachment files.]

- - - - -

For journal use only: PONEDEC3
